# Functions of the MRE11 complex in the development and maintenance of oocytes

**DOI:** 10.1007/s00412-015-0535-8

**Published:** 2015-08-01

**Authors:** Akiko Inagaki, Ramon Roset, John H. J. Petrini

**Affiliations:** Molecular Biology Program, Memorial Sloan-Kettering Cancer Center, New York, NY 10021 USA; Weill Graduate School of Medical Sciences, Cornell University, New York, NY 10021 USA; Institut de Recerca Biomèdica de Lleida, 25198 Lleida, Spain

## Abstract

The MRE11 complex (MRE11, RAD50, and NBS1) is a central component of the DNA damage response, governing both double-strand break repair and DNA damage response signaling. To determine the functions of the MRE11 complex in the development and maintenance of oocytes, we analyzed ovarian phenotypes of mice harboring the hypomorphic *Mre11*^*ATLD1*^ allele. *Mre11*^*ATLD1/ATLD1*^ females exhibited premature oocyte elimination attributable to defects in homologous chromosome pairing and double-strand break repair during meiotic prophase. Other aspects of meiotic progression, including attachment of telomeres to the nuclear envelope and recruitment of RAD21L, a component of the meiotic cohesin complex to the synaptonemal complex, were normal. Unlike *Dmc1*^*−/−*^ and *Trp13*^*Gt/Gt*^ mice which exhibit comparable defects in double-strand break repair and oocyte depletion by 5 days post-partum, we found that oocyte attrition occurred by 12 weeks in *Mre11*^*ATLD1/ATLD1*^. Disruption of the oocyte checkpoint pathway governed by *Chk2* gene further enhanced the survival of *Mre11*^*ATLD1/ATLD1*^ follicles. Together our data suggest that the MRE11 complex influences the elimination of oocytes with unrepaired meiotic double-strand breaks post-natally, in addition to its previously described role in double-strand break repair and homologous synapsis during female meiosis.

**Electronic supplementary material:**

The online version of this article (doi:10.1007/s00412-015-0535-8) contains supplementary material, which is available to authorized users.

## Introduction

Sexual reproduction requires the formation of haploid germ cells which are produced by the process of meiosis. Meiosis consists of two rounds of cell division preceded by a single phase of DNA replication. The first meiotic division (MI) is preceded by prophase during which homologous chromosomes align, pair, and recombine. Prophase is divided into five stages based on the level of homologous chromosome pairing (leptonema, zygonema, pachynema, diplonema, and diakinesis). In many species, including yeast and mammals, homologous chromosome pairing requires and is coincident with the formation and repair of meiosis-specific DNA double-strand breaks (DSBs) (reviewed in Keeney (2001)).

Meiotic DSBs are catalyzed by the dimeric topoisomerase II-like enzyme SPO11, which leaves a monomer covalently attached to the 5′ ends of the DSB end upon generation of the break (de Massy et al. 1995; Keeney and Kleckner 1995). Subsequently, MRE11 endonuclease activity releases a SPO11-coupled oligonucleotide from DSB ends to permit the resolution of meiotic DSBs by homology-directed repair (HDR) between homologous chromosomes (Farah et al. 2009; Hartsuiker et al. 2009; Milman et al. 2009; Rothenberg et al. 2009).

A critical outcome of HDR-mediated repair is the formation of crossovers. This exchange of maternal and paternal chromatid segments is required to establish physical connections between maternal and paternal chromosomes, which in turn ensures proper chromosome segregation during the first meiotic division. Only a small minority of DSBs are repaired with the formation of crossovers, but each chromosome pair must have at least one crossover to prevent missegregation and the subsequent creation of dysfunctional gametes (Petronczki et al. 2003).

DSB formation and chromosome pairing are initiated during leptonema, progress throughout zygonema, and are completed at the pachytene stage. Throughout these stages, synapsis is coordinated with the formation of the synaptonemal complex (SC), a proteinaceous structure that runs parallel to the axes of the paired homologous chromosomes (Fawcett 1956; Moses 1956). The SC consists of lateral elements along the chromosomal axes of each homolog and a central connecting element (reviewed in Yang and Wang (2009)). The formation and repair of meiotic DSBs are essential to achieve synapsis, as evidenced by the severe chromosome pairing abnormalities that are observed in mouse lacking the SPO11 enzyme and in mice that carry mutations in (meiotic) DSB-repair genes such as *Dmc1* and *Rad51C* (Kuznetsov et al. 2007; Pittman et al. 1998; Yoshida et al. 1998; Baudat et al. 2000; Romanienko and Camerini-Otero 2000).

The *Mre11* hypomorphic mutant, *Mre11*^*ATLD1*^, is one of the several *Mre11* alleles that cause the ataxia–telangiectasia like disorder (A-TLD) (Stracker and Petrini 2011). *Mre11*^*ATLD1*^ encodes a nonsense mutation at amino acid 633, causing a C-terminal truncation that deletes one of the two putative MRE11 DNA binding domains (Stracker and Petrini 2011; Stewart et al. 1999; Usui et al. 1998). Mitotic *Mre11*^*ATLD1/ATLD1*^ cells closely phenocopy ATM-deficient cells and exhibit cell cycle checkpoint defects, genome instability, and hypersensitivity to ionizing radiation (Stewart et al. 1999; Theunissen et al. 2003). These phenotypic outcomes are consistent with the role of the MRE11 complex in the activation of ATM.

Unlike males, *Mre11*^*ATLD1/ATLD1*^ females are infertile and frequently produce blastocysts that appear to die at or before the cavitation stage, indicating that the MRE11 complex is critical for early embryonic cell divisions (Theunissen et al. 2003). This prompted us to examine meiotic recombination in *Mre11*^*ATLD1*^ mice. We observed a pronounced delay in the progression of meiotic prophase and sexually dimorphic crossover behavior: Males exhibited increased and females decreased recombination (Cherry et al. 2007). In this study, we present a detailed analysis of female meiosis and oogenesis in *Mre11*^*ATLD1/ATLD1*^ mice. We observe frequent heterologous synapsis and asynapsis, persistent DSBs, reduced crossover formation, and a subsequent elimination of oocytes during folliculogenesis. The elimination of *Mre11*^*ATLD1/ATLD1*^ oocytes was CHK2 dependent as oocyte numbers were rescued in *Mre11*^*ATLD1/ATLD1*^*Chk2*^*−/*−^ females. The data presented here reveal that the functions of the MRE11 complex and CHK2 are interdependent in the response to meiotic DSBs during oogenesis and folliculogenesis.

## Results

### Premature elimination of oocytes in *Mre11*^*ATLD1/ATLD1*^ mice

We showed previously that *Mre11*^*ATLD1/ATLD1*^ females exhibit a high rate of embryonic lethality (72 % if bred with a *wild-type* male and 90 % with *Mre11*^*ATLD1/ATLD1*^ male) at embryonic day 3.5 (E3.5) (Theunissen et al. 2003). This observation indicated that oocytes produced in *Mre11*^*ATLD1/ATLD1*^ females are largely incapable of producing viable zygotes. To gain insight regarding this outcome, we examined folliculogenesis and oocyte development in *Mre11*^*ATLD1/ATLD1*^ females.

Over the course of the first 5 days after birth, oocytes become surrounded by a single layer of granulosa cells to form primordial follicles, which represent a quiescent pool of oocytes arrested at the dictyate stage, which is maintained until just before ovulation. Folliculogenesis and development of primordial follicles into mature follicles is marked by the proliferation of granulosa cells, which support the dictyate oocytes (Elvin and Matzuk 1998).

**Fig. 1:**
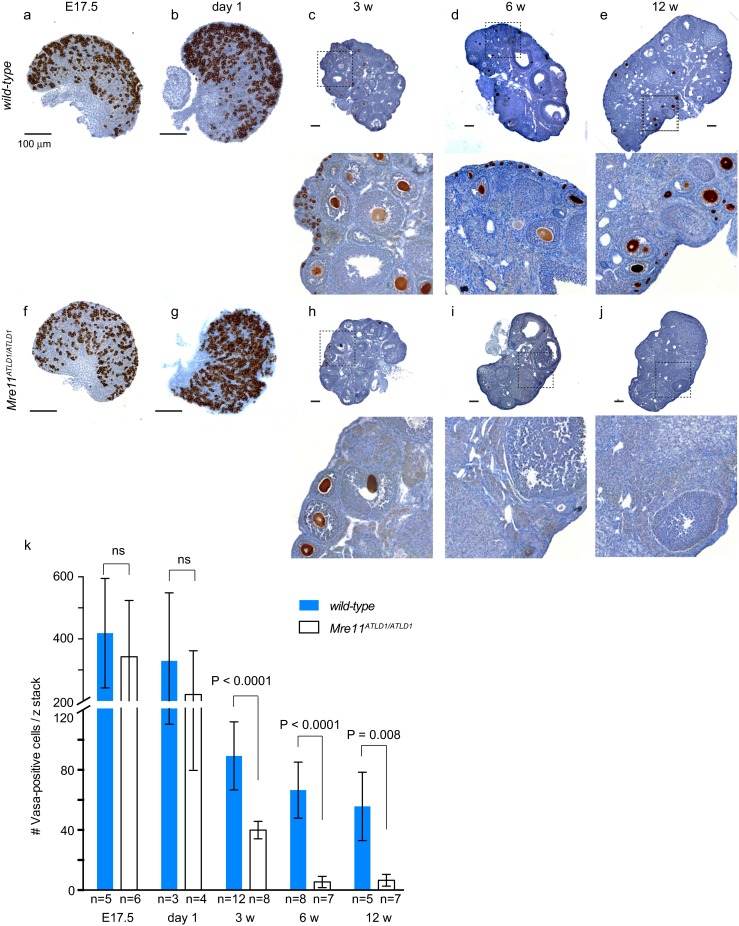
Premature elimination of *Mre11* ^*ATLD1/ATLD1*^ oocytes. **a**–**j** Representative images of anti-VASA-stained mid-ovary sections in **a**–**e** *wild-type* and **f**–**j** *Mre11* ^*ATLD1/ATLD1*^ ovaries, from E17.5 (**a** and **f**), day 1 (**b** and **g**), 3-week-old (**c** and **h**), 6-week-old (**d** and **i**), and 12-week-old (**e** and **j**). *Bar* = 100 μm. **k** Quantification of the number of follicles. *Bars* denote the average ± standard deviation (SD). Age and the number of ovaries analyzed were shown below the *x-axis. P*-value was determined by unpaired *t*-test. *Blue* and *white bars* indicate *wild-type* and *Mre11* ^*ATLD1/ATLD1*^, respectively

We assessed the number and developmental features of follicles in *Mre11*^*ATLD1/ATLD1*^ females from E17.5 to adult. Ovary sections from E17.5 and post-natal day 1 embryos were prepared and stained with VASA, a marker of oocytes (Fujiwara et al. 1994). In embryonic stage E17.5 (Fig. 1a, f) and post-natal day 1 (Fig. 1b, g), *wild-type* and *Mre11*^*ATLD1/ATLD1*^ ovaries contained similar numbers of oocytes (E17.5/*wild-type* = 419, *Mre11*^*ATLD1/ATLD1*^ = 342, *p* = 0.5. Day 1/*wild-type* = 329, *Mre11*^*ATLD1/ATLD1*^ = 221, *p* = 0.5) (Fig. 1k). TUNEL staining of post-natal day 1 mice from both genotypes was unremarkable (data not shown), suggesting that apoptotic attrition of oocytes was not occurring at this stage.

*Mre11*^*ATLD1/ATLD1*^ ovaries exhibited a precipitous loss of follicles, with complete depletion observed by 12 weeks. At 3 weeks, the number of primordial follicles declined by 12-fold in *Mre11*^*ATLD1/ATLD1*^ (*wild-type* = 53.9, *Mre11*^*ATLD1/ATLD1*^ = 4.4), while the number of mature follicles was unchanged in *wild-type* and *Mre11*^*ATLD1/ATLD1*^ (*wild-type* = 35.4, *Mre11*^*ATLD1/ATLD1*^ = 35.3) (Fig. 1c, h). By 6 weeks, attrition of mature follicles was also observed in *Mre11*^*ATLD1/ATLD1*^ (Fig. 1i, k) relative to *wild-type* (Fig. 1d, k) (*wild-type* = 66.5, *Mre11*^*ATLD1/ATLD1*^ = 5.4). At 12 weeks, a single follicle was detected in *Mre11*^*ATLD1/ATLD1*^ (Fig. 1j, and enlarged image), in contrast to *wild-type* ovaries in which mature follicles were readily detected (Fig. 1e, k). There were several corpora lutea observed at this point in *Mre11*^*ATLD1/ATLD1*^, suggesting the presence of antecedent mature oocytes. These data indicated that similar numbers of oocytes are produced in *wild-type* and *Mre11*^*ATLD1/ATLD1*^ females, but experience accelerated attrition in the latter context. This depletion likely underlies the infertility of *Mre11*^*ATLD1/ATLD1*^ females.

### Failure in homologous synapsis during meiotic prophase I in *Mre11*^*ATLD1/ATLD1*^

The failure to repair DSBs induced by SPO11 during meiotic prophase I has previously been shown to cause oocyte attrition. For example, in *Atm*^*−/−*^ and *Dmc1*^*−/−*^ ovaries, oocytes are lost within 5 days post-partum (Di Giacomo et al. 2005). Female meiosis is initiated during embryonic development around E13.5. Oocytes progress through the leptotene and zygotene stages, and at E17.5, the vast majority of oocytes reached the pachytene stage, where homologous chromosomes pair and form the SC. Complete synapsis is essential for proper meiotic development (reviewed in Inagaki et al. (2010)).

**Fig. 2:**
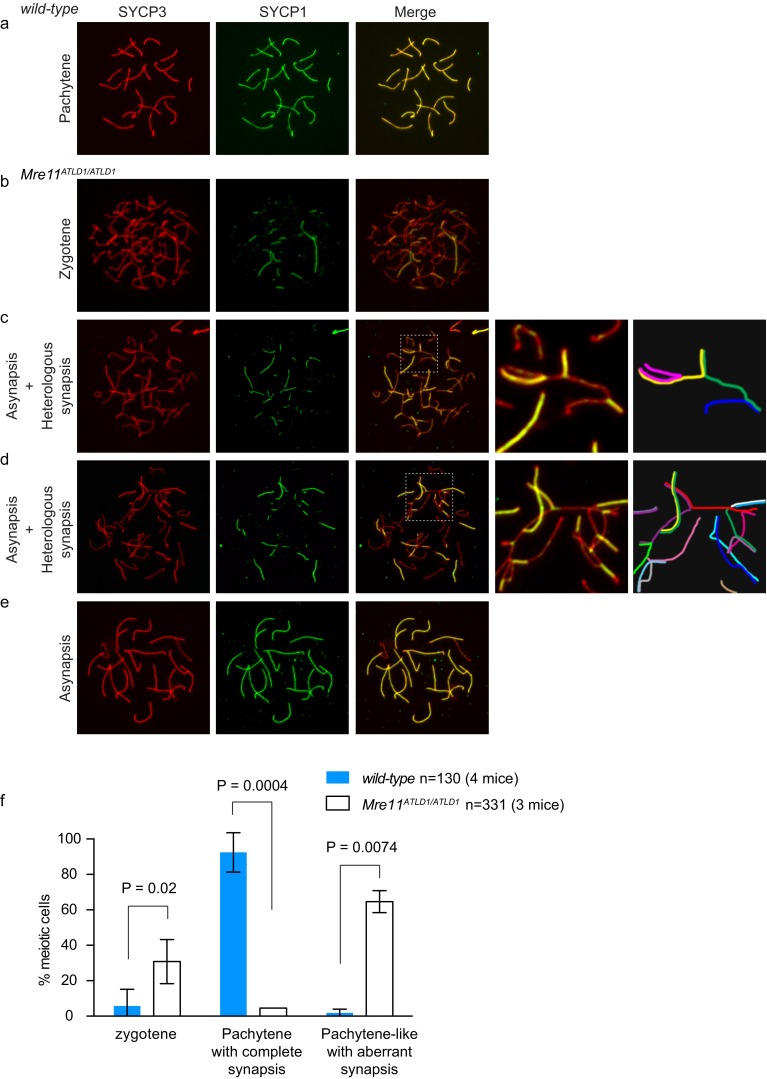
Synaptonemal complex assembly defects in *Mre11* ^*ATLD1/ATLD1*^ oocytes. **a**–**e** Representative images of double-staining with SYCP3 (*red*) and SYCP1 (*green*) in **a** *wild-type* and **b**–**e** *Mre11* ^*ATLD1/ATLD1*^ oocyte nuclei at E17.5. **c** and **d** Enlarged images and drawings were shown in the next to the merged panel. In the drawings, each SYCP3 staining was marked with different colors. **f** Quantification of the distribution of meiotic prophase substages in *wild-type* and *Mre11* ^*ATLD1/ATLD1*^ oocytes. The number of oocytes and mice analyzed were indicated. *Bars* denote the average ± SD. *P*-value was determined by unpaired *t*-test. *Blue* and *white bars* indicate *wild-type* and *Mre11* ^*ATLD1/ATLD1*^, respectively

We examined the state of meiotic progression in *Mre11*^*ATLD1/ATLD1*^ at time points corresponding to *wild-type* pachynema (E17.5) and dictyate stage (newborn). Consistent with our previous analyses (Cherry et al. 2007), *Mre11*^*ATLD1/ATLD1*^ exhibited only 5 % complete synapsis, while 31 % of oocytes were still in zygonema (Fig. 2b, f), indicating that *Mre11*^*ATLD1/ATLD1*^ shows a delay in meiotic progression. We noted that 65 % of *Mre11*^*ATLD1/ATLD1*^ nuclei exhibited pachytene-like stage with heterologous synapsis (Fig. 2c, d; enlarged drawings) or partial asynapsis (Fig. 2e) was observed. We noted that partial heterologous synapsis often appeared to initiate from one of the telomeric ends (Fig. 2c, d, enlarged drawings). The degree of asynapsis was variable from two to ten homologous chromosomes (Fig. 2e). *Mre11*^*ATLD1/ATLD1*^ nuclei often exhibited both classes of aberrancy (Fig. 2c, d). These data indicate that the MRE11 complex promotes homologous synapsis in female meiosis.

### Defective DSB processing in the *Mre11*^*ATLD1/ATLD1*^ oocytes

**Fig. 3:**
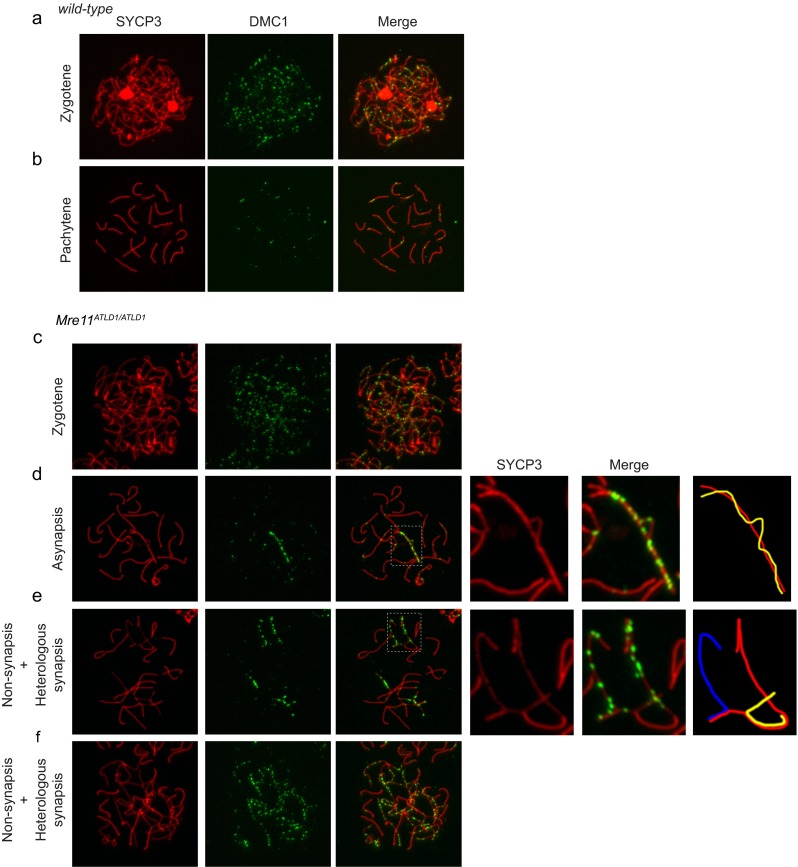
Presence of SPO11-induced meiotic DSBs in *Mre11* ^*ATLD1/ATLD1*^ oocytes. **a**–**f** Representative images of double-staining with SYCP3 (*red*) and DMC1 (*green*) in (**a** and **b**) *wild-type* and (**c**-**f**) *Mre11* ^*ATLD1/ATLD1*^ oocyte nuclei at **a** E15.5 and **b**–**f** E17.5. Substages of the meiotic prophase are shown next to the panel. **d** and **e** Enlarged images and drawings were shown in the next to the merged panel. In the drawings, each SYCP3 staining was marked with different colors. Over 100 nuclei were analyzed from three independent mice

Aberrant synapses such as those observed in *Mre11*^*ATLD1/ATLD1*^ mice are also observed in SPO11-deficient mice (Baudat et al. 2000; Romanienko and Camerini-Otero 2000). However, SPO11-induced DSBs are formed at normal levels in *Mre11*^*ATLD1/ATLD1*^ in leptonema quantified with the number of DMC1 foci (*wild-type* = 141 ± 2.4, *Mre11*^*ATLD1/ATLD1*^ = 174 ± 16.5; *p* = 0.07 (Online Resource 1). DMC1 foci persisted in *Mre11*^*ATLD1/ATLD1*^ pachytene-like oocytes and were generally restricted to asynapsed chromosomal segments (Fig. 3d–f, enlarged images), reflecting compromised DSB repair.

**Fig. 4:**
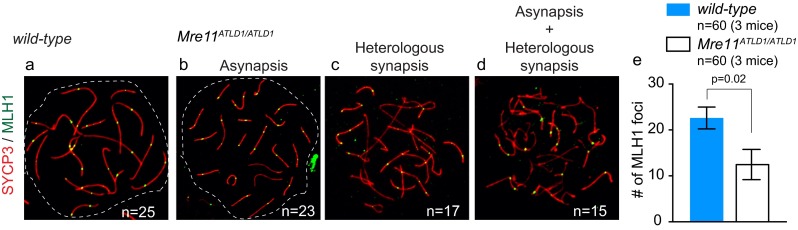
Reduced crossovers in *Mre11* ^*ATLD1/ATLD1*^ oocytes. **a**–**d** Representative images of double-staining with SYCP3 (*red*) and MLH1 (*green*) in **a** *wild-type* and **b**–**d** *Mre11* ^*ATLD1/ATLD1*^ oocyte nuclei at E17.5. The *number* of the MLH1 foci is indicated in the images. The *white dashed lines* in **a** and **b** indicate the boundary of the nucleus. **e** Quantification of the MLH1 foci in *wild-type* and *Mre11* ^*ATLD1/ATLD1*^ oocytes. *Bars* denote the average ± SD. *P*-value was determined by unpaired *t*-test. The *number* of oocytes and mice analyzed were indicated. *Blue* and *white bars* indicate *wild-type* and *Mre11* ^*ATLD1/ATLD1*^, respectively

MLH1 foci in *Mre11*^*ATLD1/ATLD1*^ pachytene-like oocytes were reduced relative to *wild-type* pachytene oocytes (*wild-type* = 23.8 ± 2.3, *Mre11*^*ATLD1/ATLD1*^ = 13.3 ± 4.6; *p* = 0.02, Fig. 4e) consistent with our previous data (Fig. 4a–e) (Cherry et al. 2007). In contrast to our previous study, we quantified all oocytes with MLH1 foci. As some oocytes exhibited severely synaptonemal complex defects (Online Resource 2), the number of MLH1 foci compared to our previous data is reduced (Cherry et al. 2007). No MLH1 foci were detected on asynapsed chromosomes, whereas the most of partially paired chromosomes exhibited MLH1 foci in *Mre11*^*ATLD1/ATLD1*^ (Fig. 4c, d), suggesting marking positions of crossovers between heterologous chromosomes since 65 % of *Mre11*^*ATLD1/ATLD1*^ nuclei exhibited pachytene-like stage with heterologous synapsis or partial asynapsis (Fig. 2).

**Fig. 5:**
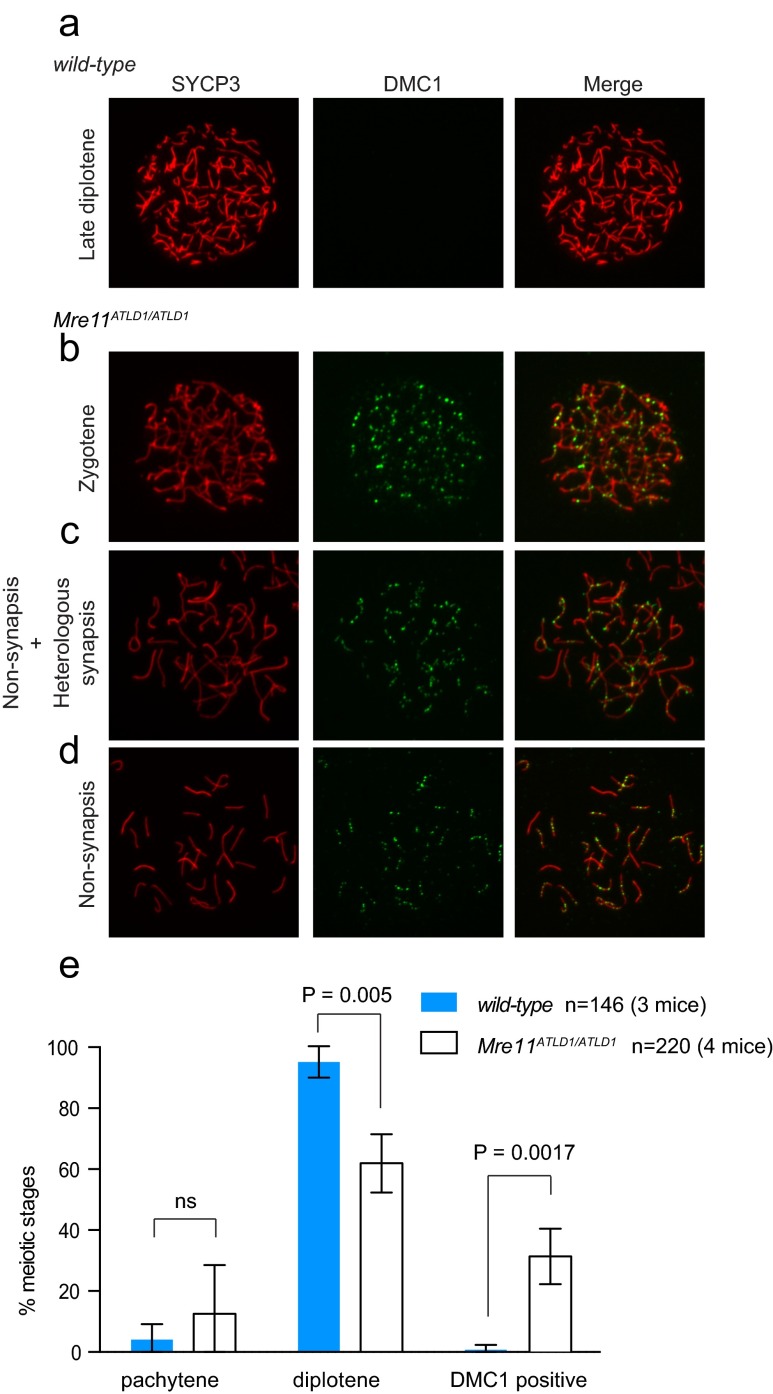
Persistent meiotic DSBs in *Mre11* ^*ATLD1/ATLD1*^ newborn oocytes. **a**–**d** Representative images of double-staining with SYCP3 (*red*) and DMC1 (*green*) in **a** *wild-type* and **b**–**d** *Mre11* ^*ATLD1/ATLD1*^ oocyte nuclei from newborn. **e** Quantification of the distribution of meiotic prophase substages and DMC1 positive nuclei in *wild-type* and *Mre11* ^*ATLD1/ATLD1*^ oocytes. *Bars* denote the average ± SD. *P*-value was determined by unpaired *t*-test. The number of oocytes and mice analyzed were indicated. *Blue* and *white bars* indicate *wild-type* and *Mre11* ^*ATLD1/ATLD1*^, respectively

In newborn mice, fewer than 2 % of *wild-type* nuclei contained DMC1 foci (Fig. 5a, e). In contrast, more than 30 % of nuclei were positive with DMC1 foci in *Mre11*^*ATLD1/ATLD1*^ (Fig. 5e), indicating the persistence of DSBs, that exhibited hallmarks of zygotene or pachytene-like stages (Fig. 5b–d). Consistent with the presence of DSBs in oocytes of newborn *Mre11*^*ATLD1/ATLD1*^ mice, 80 % of *Mre11*^*ATLD1/ATLD1*^ oocytes were γH2AX-positive compared with 10 % of *wild-type* oocytes (Online Resource 3). These results suggest that unrepaired DSBs and aberrant synapsis underlie the premature depletion of follicles in *Mre11*^*ATLD1/ATLD1*^ ovaries.

### Normal telomere attachment and RAD21L recruitment in *Mre11*^*ATLD1/ATLD1*^ oocytes

**Fig. 6:**
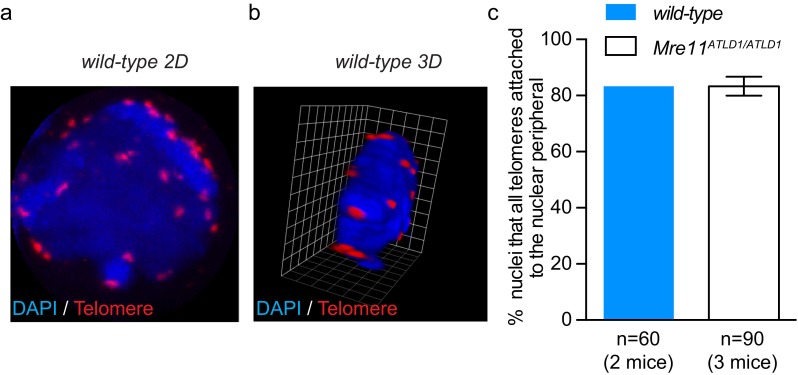
Normal attachment of telomeres to the nuclear envelope in *Mre11* ^*ATLD1/ATLD1*^ oocytes. **a** and **b** Representative images of telomere FISH (*red*) in *wild-type* oocyte nucleus at E17.5 in **a** two-dimension and **b** three-dimension. **c** Quantification of telomere attachment to the nuclear envelop in *wild-type* and *Mre11* ^*ATLD1/ATLD1*^ oocytes. Shown is the percentage of the oocytes that show all detectable telomere FISH signals attach to the nuclear envelop. The *number* of oocytes and mice analyzed were shown below the *x-axis. Bars* denote the average ± SD. *Blue* and *white bars* indicate *wild-type* and *Mre11* ^*ATLD1/ATLD1*^, respectively

Two additional possible sequelae of MRE11 complex hypomorphism that could account for oocyte attrition were assessed: defective telomere attachment and impaired cohesin recruitment. Attachment of telomeres to the nuclear envelope from early leptonema to pachynema is required for homologous chromosome pairing, SC formation, and folliculogenesis (Ding et al. 2007). We examined whether the aberrant synapsis observed in *Mre11*^*ATLD1/ATLD1*^ was attributed to irregular localization of telomeres. Fluorescence in situ hybridization (FISH) analysis of telomeres (Tel-FISH) in cryo-sections of embryonic E17.5 ovaries was carried out. Three-dimensional image reconstructions revealed that telomeric signals were confined to the nuclear periphery (Fig. 6a, b). Both *wild-type* and *Mre11*^*ATLD1/ATLD1*^ exhibited all Tel-FISH signals that were localized at the nuclear peripheral in more than 80 % of the oocytes (Fig. 6c). In the remaining 20 % of the nuclei, a single Tel-FISH signal was mislocalized and appeared within the nucleus. These data excluded the possibility of irregular telomere localization as a cause of aberrant synapsis in *Mre11*^*ATLD1/ATLD1*^.

MRE11 complex-dependent recruitment of cohesin complexes has been described in yeast and human cell lines (Kim et al. 2002; Tittel-Elmer et al. 2012). In meiotic cells, cohesin promotes homology search and recombination between homologous chromatids by assembly of the SC (reviewed in Inagaki et al. (2010)). Deletion of meiosis-specific cohesin subunits in mice (*Rec8*^*−/−*^, *Smc1b*^*−/−*^ and *Rad21L*^*−/−*^) showed defects in synapsis and infertility (Bannister et al. 2004; Revenkova et al. 2004; Xu et al. 2005) and premature reproductive senescence in *Rad21L*^*−/−*^ females associated with a defect in primordial follicles (Herran et al. 2011).

**Fig. 7:**
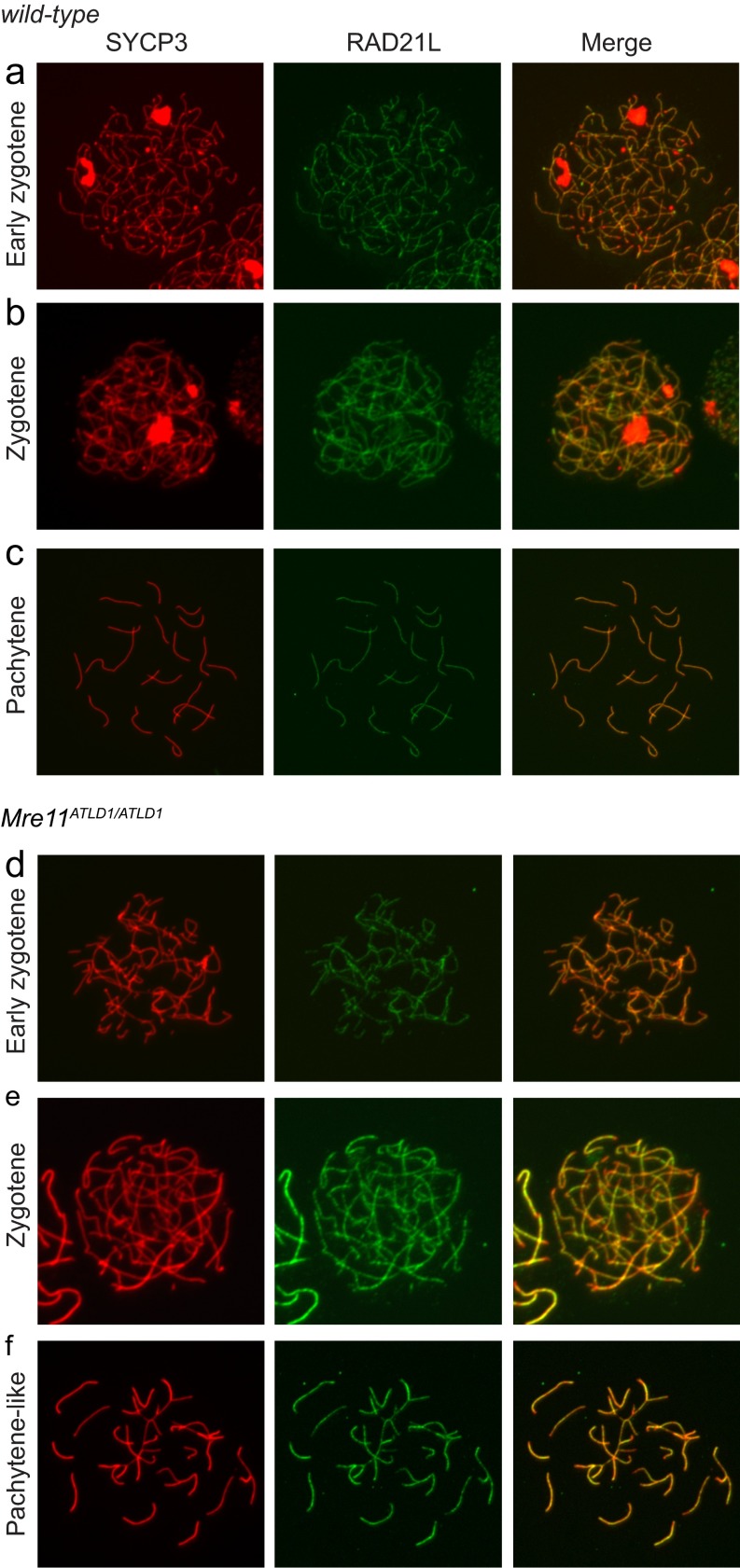
No detectable difference of RAD21L recruitment to axial elements in *Mre11* ^*ATLD1/ATLD1*^ oocytes. **a**–**f** Representative images of double-staining with SYCP3 (*red*) and RAD21L (*green*) in **a**–**c** *wild-type* and **d**–**f** *Mre11* ^*ATLD1/ATLD1*^ oocyte nuclei at (**a**, **b**, **d**, and **e**) E15.5 and (**c** and **f**) E17.5. Substages of the meiotic prophase are shown next to the panel. More than 300 nuclei were analyzed from three independent mice

Accordingly, we examined recruitment of RAD21L to axial elements in *Mre11*^*ATLD1/ATLD1*^. RAD21L was localized along the SYCP3 from early zygonema to late pachynema at synapsed and asynapsed parts, and the localization was essentially identical in *wild-type* and *Mre11*^*ATLD1/ATLD1*^ (Fig. 7a–f). Despite aberrant synapses in *Mre11*^*ATLD1/ATLD1*^ pachytene-like nuclei, RAD21L was observed at paired axial element regions (Fig. 7f), suggesting that aberrant synapsis observed in *Mre11*^*ATLD1/ATLD1*^ oocytes was not due to the irregular localization of RAD21L.

### CHK2-dependent elimination of *Mre11*^*ATLD1/ATLD1*^ oocytes

CHK2 governs a p53- and p63-dependent DNA damage checkpoint response which promotes elimination of oocytes harboring unrepaired meiotic DSBs ( Bolcun-Filas et al. 2014). Previous studies of *Mre11*^*ATLD1/ATLD1*^*Chk2*^*−/−*^ mice have shown that CHK2 suppresses the oncogenic potential of DNA damage arising in S phase. This CHK2 function was revealed by the observation that CHK2 deficiency in *Mre11*^*ATLD1/ATLD1*^ mice—which exhibit pronounced chromosome fragility during DNA replication—leads to a broad spectrum of tumorigenesis (Stracker et al. 2008). This observation indicated that CHK2 is required to mitigate the effects of DNA damage accumulating as a result of MRE11 complex hypomorphism.

**Fig. 8:**
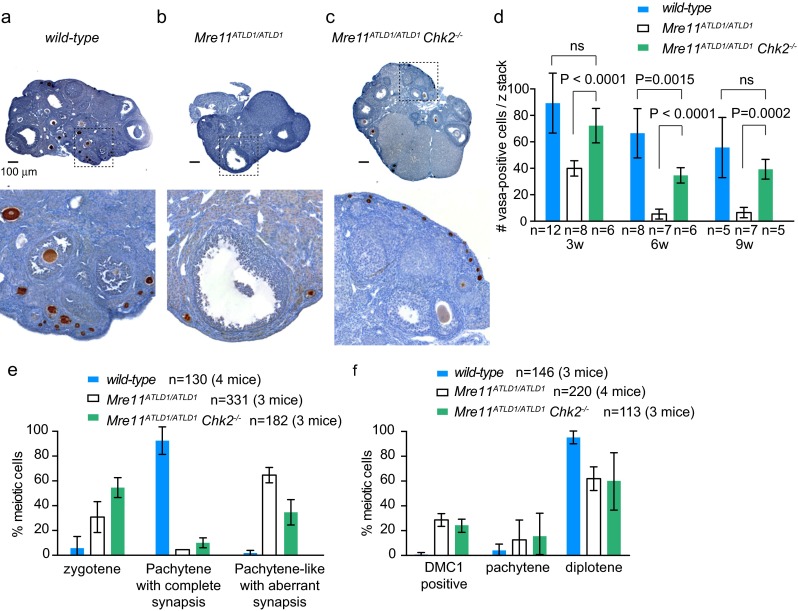
Restored follicles by depletion of *Chk2* in adult *Mre11* ^*ATLD1/ATLD1*^ ovaries. (**a**-**c**) Representative images of anti-VASA-stained mid-ovary sections in (**a**) *wild-type*, (**b**) *Mre11* ^*ATLD1/ATLD1*^, and (**c**) *Mre11* ^*ATLD1/ATLD1*^ *Chk2* ^*−/−*^ at 9-week-old. Enlarged images show the restored number of primordial follicles by *Chk2* deletion. Bar = 100 μm. (**d**) Quantification of the number of follicles. The number of ovaries analyzed was indicated below the x-axis. (**e**) Quantification of the distribution of meiotic prophase substages at E17.5 in *wild-type*, *Mre11* ^*ATLD1/ATLD1*^ and *Mre11* ^*ATLD1/ATLD1*^ *Chk2* ^*−/−*^. Pachytene stages are classified to complete and aberrant synapsis. (**f**) Quantification of the distribution of meiotic prophase substages and DMC1 positive nuclei from newborn oocytes in *wild-type*, *Mre11* ^*ATLD1/ATLD1*^ and *Mre11* ^*ATLD1/ATLD1*^ *Chk2* ^*−/−*^. (**d**-**f**) Bars denote the average ± SD. *P*-value was determined by unpaired *t*-test. The number of nuclei and mice analyzed were indicated. Blue, white and green bars indicate *wild-type*, *Mre11* ^*ATLD1/ATLD1*^ and *Mre11* ^*ATLD1/ATLD1*^ *Chk2* ^*−/−*^, respectively

A similar relationship appears to be operative in meiotic tissue. CHK2 deficiency markedly enhanced the survival of developing follicles in 3-, 6-, and 9-week-old *Mre11*^*ATLD1/ATLD1*^ ovaries as the total number of follicles in *Mre11*^*ATLD1/ATLD1*^*Chk2*^*−/−*^ were similar to the *wild-type* level (*wild-type* = 58, *Mre11*^*ATLD1/ATLD1*^ = 6, *Mre11*^*ATLD1/ATLD1*^*Chk2*^*−/−*^ = 44) (Fig. 8a–d). This outcome supports the conclusion that oocyte attrition in *Mre11*^*ATLD1/ATLD1*^ is caused by CHK2-dependent elimination of oocytes that harbor unresolved meiotic DSBs. As expected, CHK2 deficiency had no effect on the disrupted synaptic phenotype in *Mre11*^*ATLD1/ATLD1*^ oocytes (Fig. 8e, f), and the fertility of *Mre11*^*ATLD1/ATLD1*^ was not rescued (data not shown).

## Discussion

The *Mre11*^*ATLD1*^ allele is one of several *Mre11* alleles underlying the human A-TLD in which attenuated ATM activation essentially phenocopies ATM deficiency (Stracker and Petrini 2011). In this study, we used the *Mre11*^*ATLD1*^ allele to examine the MRE11 complex and ATM-dependent DNA damage response in female meiosis. Meiotic progression was delayed in *Mre11*^*ATLD1/ATLD1*^ mice and associated with failure in homologous synapsis, persistence of DSBs, and reduced crossover frequency, consistent with defective homologous recombination during meiotic prophase (Cherry et al. 2007).

Presumably due to the presence of multiple persistent DSBs in meiotic prophase, *Mre11*^*ATLD1/ATLD1*^ oocytes were arrested in a pachytene-like stage, with normal number of oocytes present in newborn ovaries. However, we observed attrition of oocytes from adult ovaries, as observed in *Dmc1*^*−/−*^ mutants (Di Giacomo et al. 2005). In *Dmc1*^*−/−*^ mutants, CHK2 deficiency sharply reduced oocyte attrition, without rescuing fertility ( Bolcun-Filas et al. 2014). Unlike *Dmc1*^*−/−*^ mice in which precipitous attrition is observed (Di Giacomo et al. 2005), oocyte attrition in *Mre11*^*ATLD1/ATLD1*^ occurred gradually, suggesting that the MRE11 complex influences the rate of post-natal oocyte attrition. Although that mechanism is largely dependent on ATR and CHK2 (Pacheco et al. 2015; Bolcun-Filas et al. 2014), the rate of attrition in *Mre11*^*ATLD1/ATLD1*^ raises the possibility that ATM may also influence CHK2 in the oocyte surveillance pathway. Collectively, these data underscore the importance of the MRE11 complex for the development and maintenance of oocytes.

### The MRE11 complex in meiotic DSB repair and homologous synapsis

Meiotic prophase in *Mre11*^*ATLD1/ATLD1*^ oocytes was characterized by asynapsis and heterologous synapsis (Fig. 2c–f). As homologous synapsis depends on meiotic recombination (Bolcun-Filas and Schimenti 2012), such impaired homologous synapsis may be attributed to compromised DSB repair in *Mre11*^*ATLD1/ATLD1*^ oocytes. The repair defects associated with the *Mre11*^*ATLD1*^ allele are likely to synergize with the reduction of ATM activity. For example, *Mre11*^*ATLD1/ATLD1*^ spermatocytes exhibited a twofold increase in SPO11–oligonucleotide complexes compared to wild-type spermatocytes (Pacheco et al. 2015). If a similar increase were to occur in oocytes, the manifestations of defective DSB repair, including impaired synapsis, would likely be more pronounced. However, we did not observe increased DMC1 foci in *Mre11*^*ATLD1/ATLD1*^ leptotene oocytes that argues against this interpretation (Online resource 1), although we cannot exclude the possibility that the transient nature of leptonema limits our ability to reliably determine smaller differences in cytological outcomes. It is clear, in any case from the persistence of RAD51 and DMC1 in pachynema, that DSB repair is defective in *Mre11*^*ATLD1/ATLD1*^ oocytes (Fig. 3c–f and Cherry et al. (2007)).

### Meiotic DNA damage response leads to follicle elimination in post-natal ovaries

Genetic analyses suggest the existence of distinct checkpoints for DNA damage, synapsis, and crossover formation in oocytes. The data presented herein suggest that the process of oocyte elimination is influenced by the MRE11 complex. Oocytes showing persistent DSBs during meiotic prophase such as *Trip13*^*Gt/Gt*^ (normal synapsis) (Li and Schimenti 2007), *Rec8*^*−/−*^ (Xu et al. 2005) and *Sun1*^*−/−*^ (Ding et al. 2007) (asynapsis) or *Dmc1*^*−/−*^ and *Atm*^*−/−*^ (asynapsis and heterologous synapsis) (Di Giacomo et al. 2005) exhibit follicular elimination by 5 days post-partum. *Spo11*^*−/−*^ oocytes in which DSBs are not formed exhibit failure in synapsis (heterologous synapsis and asynapsis) but persist in follicles until 6 weeks post-partum (Di Giacomo et al. 2005). SPO11 deficiency is epistatic to *Dmc1*^*−/−*^ and *Atm*^*−/−*^ with respect to oocyte attrition (Di Giacomo et al. 2005), suggesting that DSB formation as well as the ensuing synapsis and repair are the events that determine oocyte elimination in post-natal ovaries. This is in contrast to *Mlh1*^*−/−*^ oocytes which do not exhibit follicular attrition. In that setting, DSB formation, DSB repair, and synapsis are normal, but crossover formation does not occur, resulting in infertility (Edelmann et al. 1996). Unrepaired DSBs are abundant in *Mre11*^*ATLD1/ATLD1*^ oocytes, and gross defects in synapsis are evident, yet the elimination of follicles occurred at 12 weeks post-partum, markedly later than the examples above. We propose two distinct hypotheses based on this observation.

We interpret this observation to reflect the contribution of the MRE11 complex to promoting the elimination of oocytes with synapsis and DSB repair defects. The oocyte elimination pathway depends on ATR and CHK2 ( Bolcun-Filas et al. 2014). Accordingly, inactivation of CHK2 in *Trip13*^*Gt/Gt*^, *Dmc1*^*−/−*^, *Atm*^*−/−*^ (Bolcun-Filas et al. 2014), *Mre11*^*ATLD1/ATLD1*^, and *Rad50*^*+/46*^, which harbors a mutation in the Rad50 hook domain and exhibits ovarian atrophy (Roset et al. 2014) (Online Resource 4b and c), suppresses the attrition of defective oocytes. The slower rate of attrition in *Mre11*^*ATLD1/ATLD1*^ ovaries could reflect that the MRE11 complex influences ATR activation, as has been suggested previously (Duursma et al. 2013; Shiotani et al. 2013). Alternatively, as ATM activation is compromised but not abolished in *Mre11*^*ATLD1/ATLD1*^, the data may reveal that ATM contributes to the pathway as well. In this scenario, we speculate that complete loss of ATM is pleiotropic, whereas the ATM functional deficit in *Mre11*^*ATLD1/ATLD1*^ would selectively affect the presumptive role in oocyte elimination. Precedence for circumscribed loss of certain ATM functions in MRE11 complex mutants comes from the *Nbs1*^*∆C/∆C*^ mouse in which ATM-dependent apoptosis is selectively impaired (Stracker et al. 2007). It is notable that the *Nbs1*^*∆C/∆C*^ also revealed that ATM and CHK2 act in parallel to regulate thymocyte apoptosis, making it conceivable that if ATM has a role in oocyte depletion, it could be CHK2 independent. It must also be noted that we cannot exclude the possibility that the female meiotic DNA damage checkpoint and synapsis checkpoint function in a dose-dependent manner and that the apparently slower oocyte attrition reflects a lower level of persistent DNA damage and aberrant synapsis in *Mre11*^*ATLD1/ATLD1*^ oocyte than previously characterized mutants.

Moreover, we obtained evidence in *Rad50*^*+/46*^*Chk2*^*−/−*^ mice that CHK2 may promote depletion of oocytes harboring persistent DSBs in meiotic prophase (Online Resource 4d). Despite exhibiting follicular attrition, *Rad50*^*+/46*^ mice did not exhibit overt defects in DNA repair or homologous synapsis (Online Resource 4a and d) (Roset et al. 2014). However, in *Rad50*^*46/+*^*Chk2*^*−/−*^ double mutants, aberrant heterologous synapses were readily observed at E17.5, suggesting that as in spermatocytes (Pacheco et al. 2015), CHK2 may play a role in the surveillance of meiotic recombination in female meiotic prophase.

## Materials and methods

### Mice

*Mre11*^*ATLD1*^ and *Rad50*^*+/46*^ mice were previously described (Theunissen et al. 2003; Roset et al. 2014). *Chk2*^*−/−*^ mice were obtained from T. Mak. All mice were maintained on mixed 129/SvEv and C57BL6 background.

Mice were housed in a ventilated rack caging in a pathogen-free facility. The Institutional Animal Care and Use Committee of Memorial Sloan Kettering Cancer Center approved animal use protocols.

### Antibodies

For primary antibodies, SYCP3 (goat anti-mouse monoclonal provided by S. West), SYCP1 (goat anti-rabbit, Novus), MLH1 (goat anti-mouse monoclonal, BD Pharmingen), DMC1 (goat anti-rabbit polyclonal, Santa Cruz), and RAD21L (goat anti-mouse monoclonal provided by T. Hirano) were used. For secondary antibodies, goat anti-rabbit IgG Alexa 488/564 and goat anti-mouse Alexa IgG 488/564 (Molecular Probes) were used.

### Histological sample preparation, staining, and analysis

Tissue samples for histological analyses were fixed overnight at 4 °C in 4 % paraformaldehyde, rinsed, stored at 4 °C in 70 % ethanol, and then processed for paraffin embedding. Eight-micrometer sections were prepared, and slides were processed and stained at the Memorial Sloan Kettering molecular cytogenetics core facility for hematoxylin and eosin (H&E), TUNEL, anti-VASA, and anti-γH2AX.

H&E-stained and immunohistochemically stained slides were digitally scanned using a Mirax scanner. Anti-VASA-positive cells and anti-γH2AX-positive cells per slide were manually scored.

### Meiotic spread nuclei preparations, immunocytochemistry, and analysis

Ovaries were processed to obtain spread nuclei for immunocytochemistry. Briefly, embryonic ovaries were dissected and placed in phosphate-buffered saline (PBS), pH7.4, at room temperature. Adherent extra tissue was removed in PBS. The ovaries were placed in a hypotonic extraction buffer containing 30 mM Tris, 50 mM sucrose, 17 mM trisodium citrate dehydrate, 5 mM EDTA, 0.5 mM DTT, and 0.5 mM phnylmethylsuphonyl fluoride, pH 8.2, for 20–40 min. Subsequently, an oocyte suspension was made in 10 μl of 50 mM sucrose and 10 μl of PBS by puncturing a single ovary with needles on a clean glass slide. The cell suspension was fixed with 1 % paraformaldehyde, pH 9.2 (set by using 10 mM sodium borate pH 9.2), containing 0.15 % Triton X-100 for 1.5 h. The slides were washed twice for 2 min in 0.4 % Photoflo (Kodak) and dried at room temperature.

Spread nuclei of oocytes were stained with the antibodies mentioned above. Before incubation with antibodies, slides were washed in PBS (3×10 min), and non-specific sites were blocked with 0.5 % *w*/*v* BSA and 0.5 % *w*/*v* milk powder in PBS. Primary antibodies were diluted in 10 % *w*/*v* BSA in PBS, and incubations were held overnight at room temperature in a humid chamber. Subsequently, slides were washed (3×10 min) in PBS , blocked in 10 % *v*/*v* normal goat serum (Sigma) in blocking buffer (supernatant of 5 % *w*/*v* milk powder in PBS centrifuged at 14,000 rpm for 10 min), and incubated with secondary antibodies in 10 % normal goat serum in blocking buffer at RT for 2 h. Finally, slides were washed (3×10 min) in PBS and embedded in Prolong Gold with DAPI (invitrogen).

The number of DMC1 foci was scored using the ImageJ software (Rasband, W.S., ImageJ, U.S. National Institutes of Health, Bethesda, Maryland, USA, [http://rsb.info.nih.gov/ij/]).

## Electronic supplementary material

ESM 1 (PDF 8449 kb)
