# Supplementary material for: Functions of the MRE11 complex in the development and maintenance of oocytes
Source: Chromosoma. 2015 Aug 1;125:151–62. doi: 10.1007/s00412-015-0535-8 (PMC4734907; doi:10.1007/s00412-015-0535-8)

The Mre11 complex regulates oocyte attrition

Journal name: Chromosoma

Akiko Inagaki[1], Ramon Roset[1*] and John H.J. Petrini[1,2,3]

[1]Molecular Biology Program, Memorial Sloan-Kettering Cancer Center, New York, New York 10021, USA

[2]Weill Graduate School of Medical Sciences, Cornell University, New York, New York 10021, USA

[3]Corresponding author

Email: petrinij@mskcc.org

Phone: +1-212-639-2027

Fax: +1- 646-422-2062

[*] Current address; Institut de Recerca Biomèdica de Lleida, 25198 Lleida, Spain

Online Resource 1. Quantification of SPO11-induced DSBs in *Mre11*$^{ATLD1/ATLD1}$.

(a) Quantification of the number of DMC1 foci at E15.5. Genotypes were shown below X-axis. Twenty-five oocytes were analyzed from each mouse indicated. Bars denote the average ± standard deviation (SD). (b) Combined quantification of the number of DMC1 foci based on the data from (a). P-value was determined by unpaired t-test. Genotypes were shown below X-axis. Seventy-five oocytes were analyzed from 3 independent mice.

Online Resource 2. Reduced crossovers in *Mre11*$^{ATLD1/ATLD1}$ oocytes.

(a, b) Double-staining with SYCP3 (red) and MLH1 (green) in *Mre11*$^{ATLD1/ATLD1}$ oocyte nuclei at E17.5. The number of the MLH1 foci is indicated in the images.

Online Resource 3. Persistent DSBs in *Mre11*$^{ATLD1/ATLD1}$ newborn oocytes.

Representative images of newborn ovaries stained with γH2AX (a and b) and VASA (c and d), in sequential slices of (a and c) *wild-type* and (b and d) *Mre11*$^{ATLD1/ATLD1}$ ovaries.

Online Resource 4. Restored follicles by depletion of *Chk2* in *Rad50*$^{+/46}$ ovaries.

(a and b) Representative images of anti-VASA-stained mid-ovary sections in (a) *Rad50*$^{+/46}$, (b) *Rad50*$^{+/46}$ *Chk2*$^{-/-}$ at 9-week-old. Enlarged images show the restored number of primordial follicles by *Chk2* deletion. (c) Quantification of the number of follicles at 9 weeks. The analyzed number of ovaries was indicated below the x-axis. (d) Quantification of the percentage of E17.5 oocytes with

aberrant meiotic synapsis in *wild-type*, *Rad50*$^{+/46}$, *Chk2*$^{-/-}$, and *Rad50*$^{+/46}$ *Chk2*$^{-/-}$. Over 100 oocytes per mouse were anlyzed and the number of mice analyzed were indicated. Bars denote the average ± SD. *P*-value was determined by unpaired *t*-test. Blue, gray, green and black bars indicate *wild-type*, *Rad50*$^{+/46}$, *Chk2*$^{-/-}$, and *Rad50*$^{+/46}$ *Chk2*$^{-/-}$, respectively.

Online Resource 1

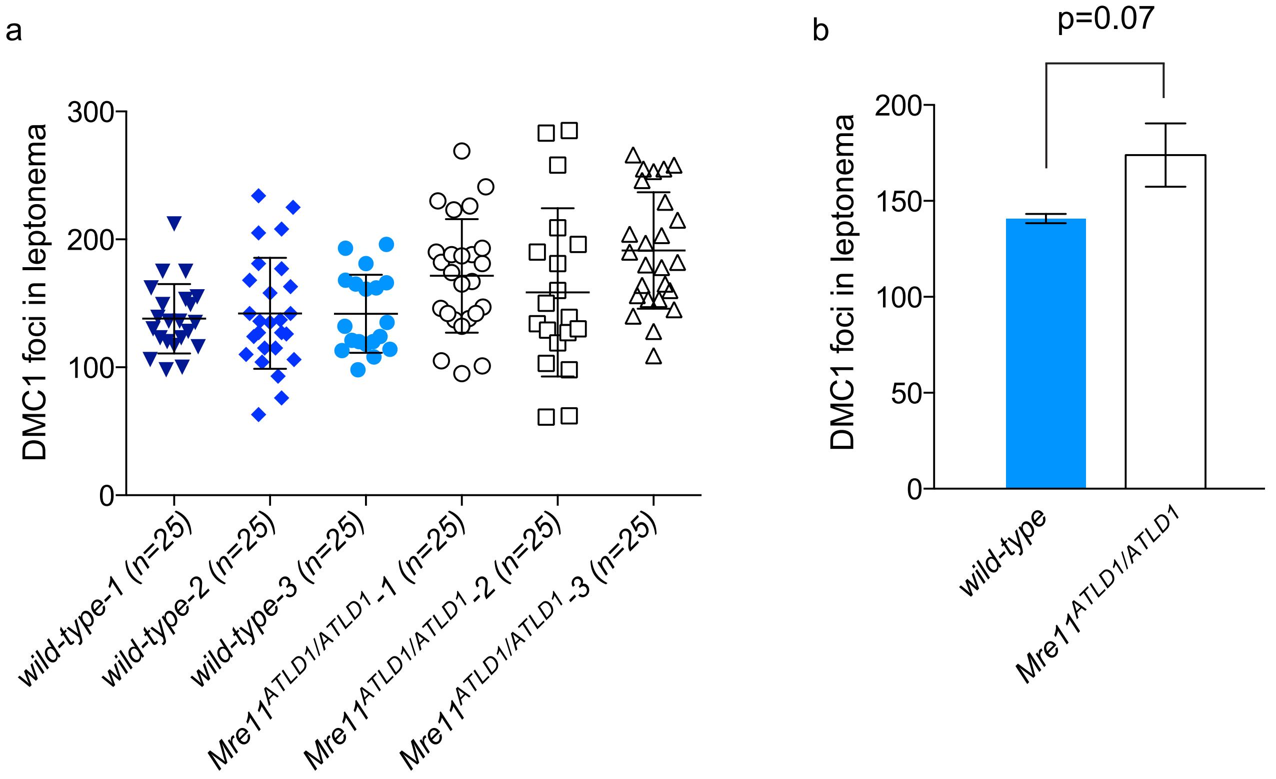

Online Resource 2

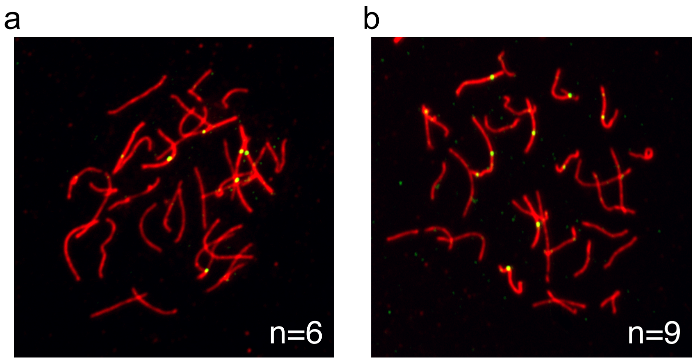

Online Resource 3

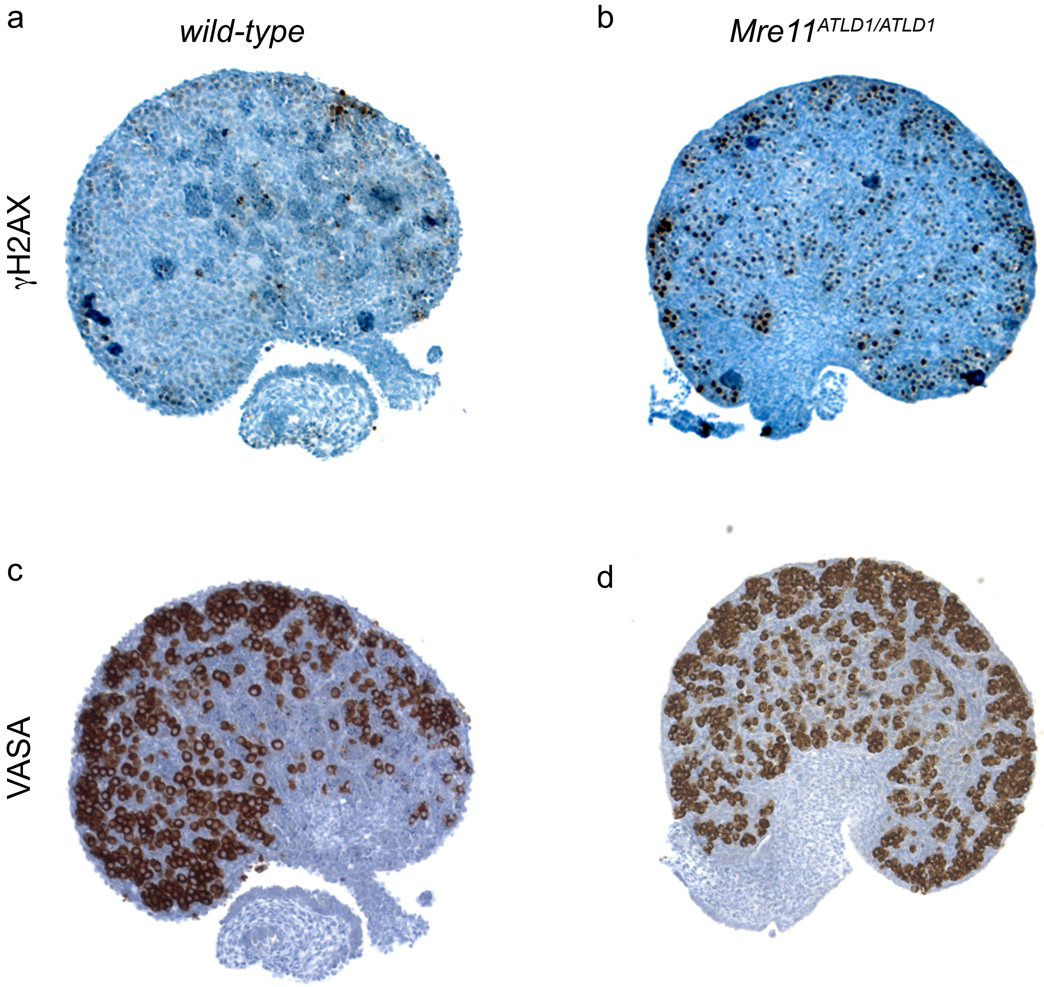

Online Resource 4

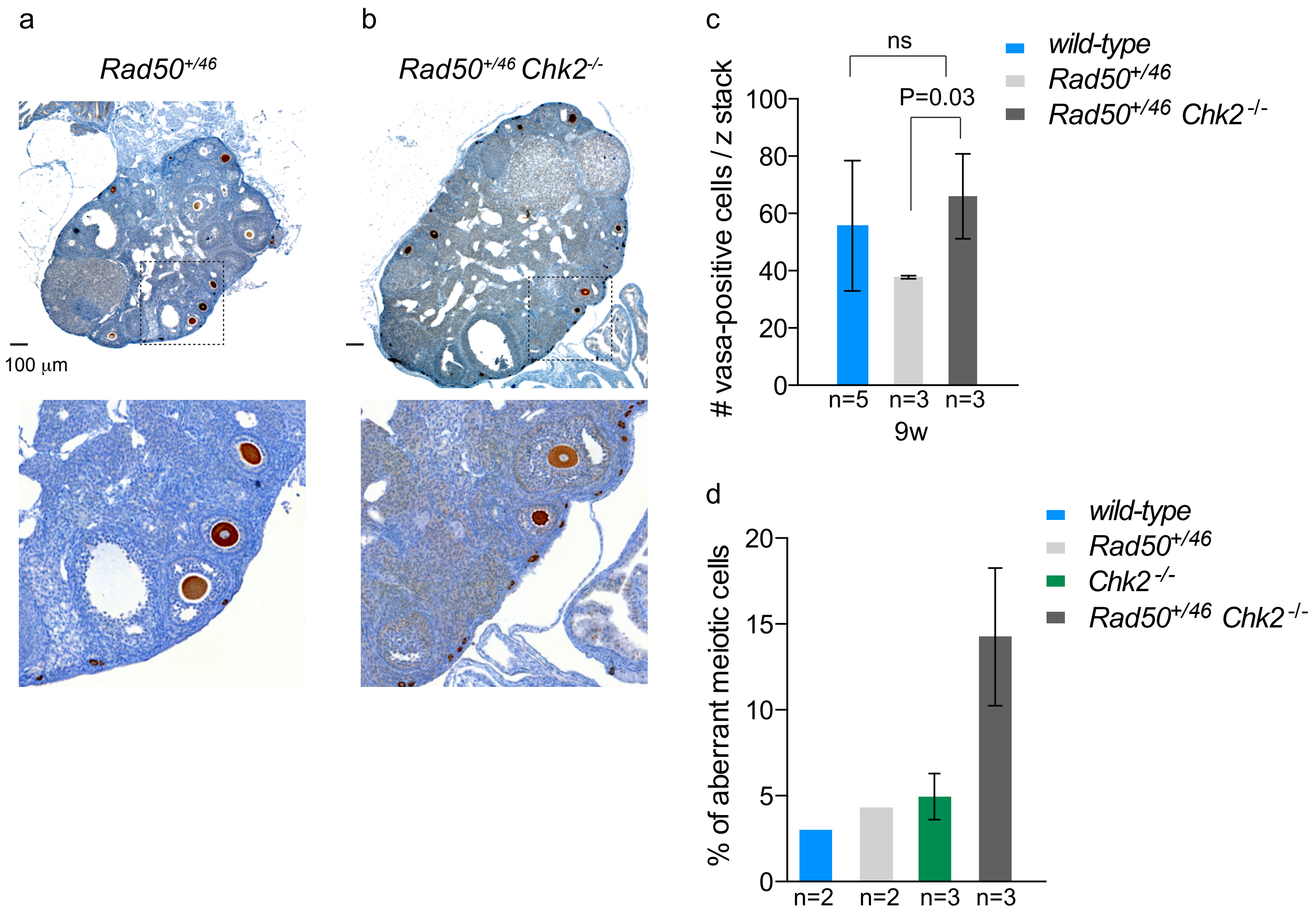

Supplement: Supplementary file 1 — (PDF 8449 kb) [file 412_2015_535_MOESM1_ESM.pdf]
